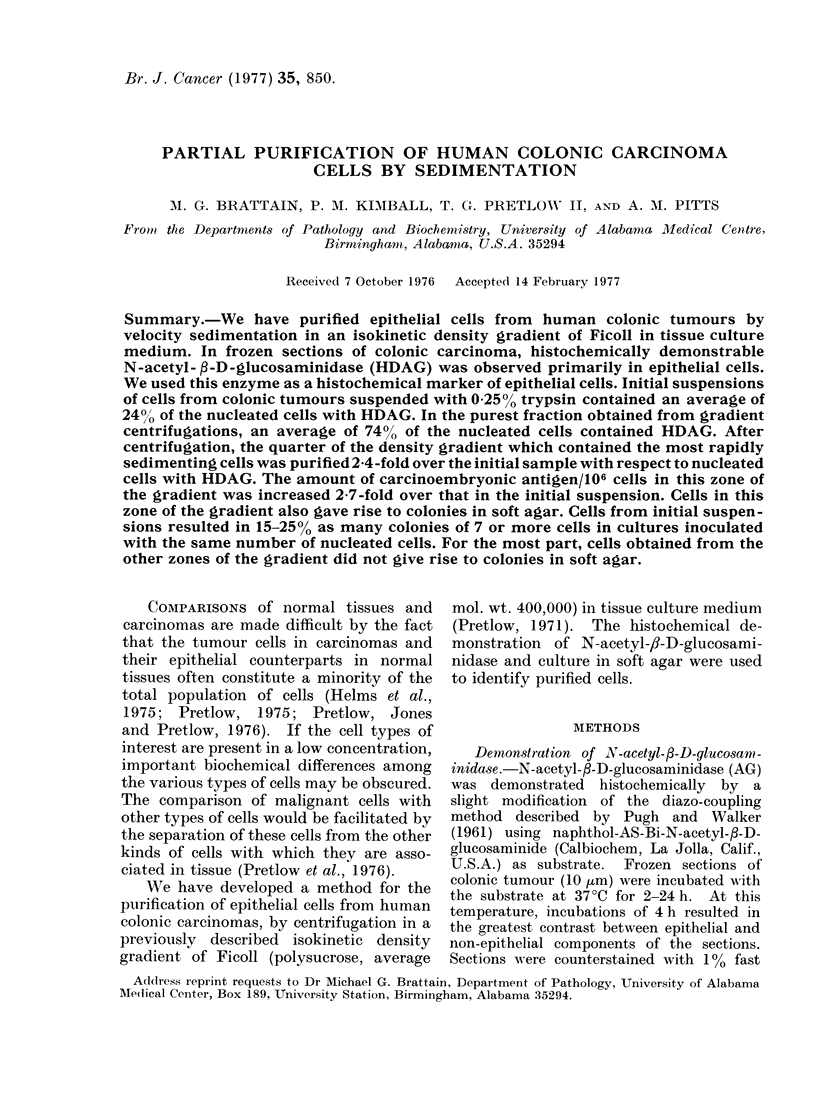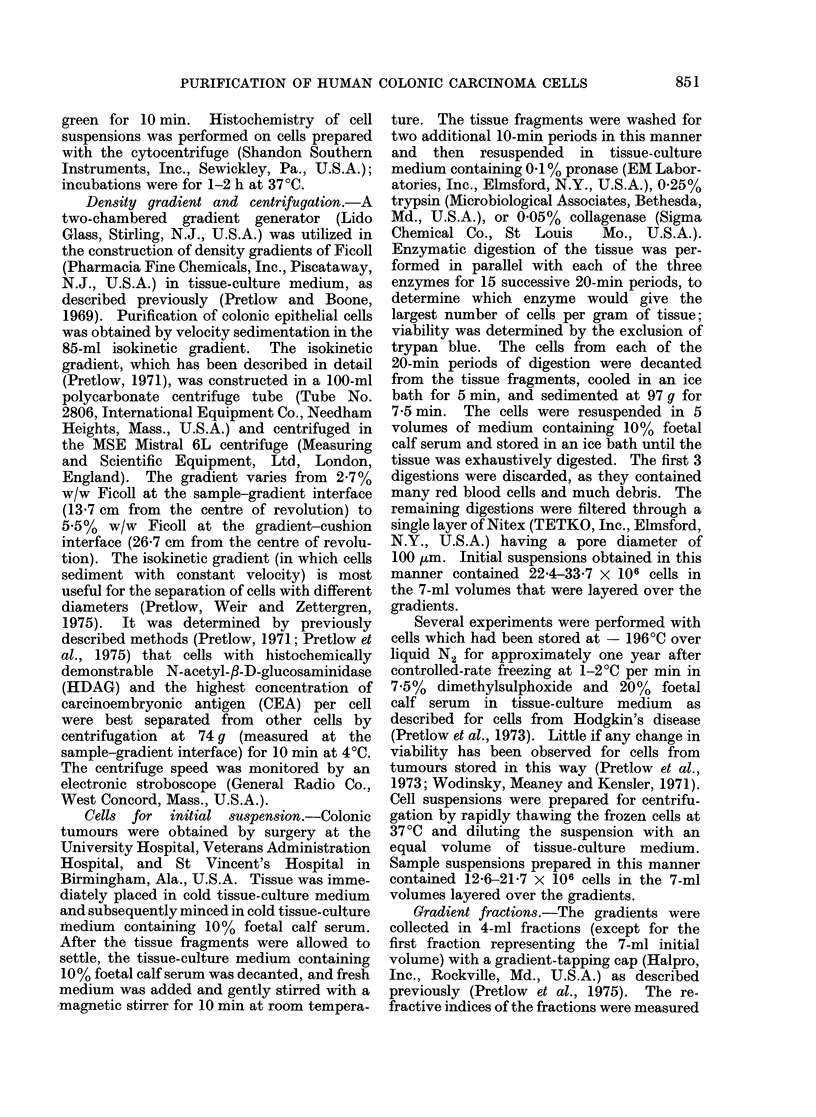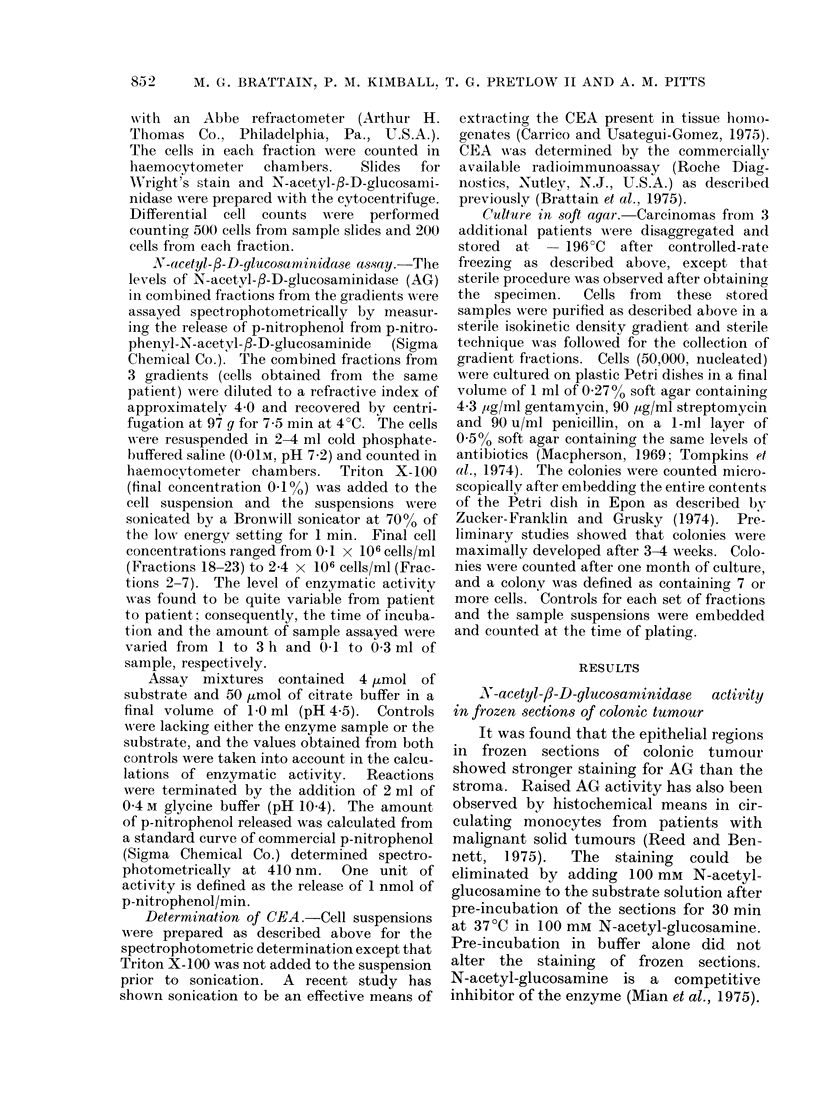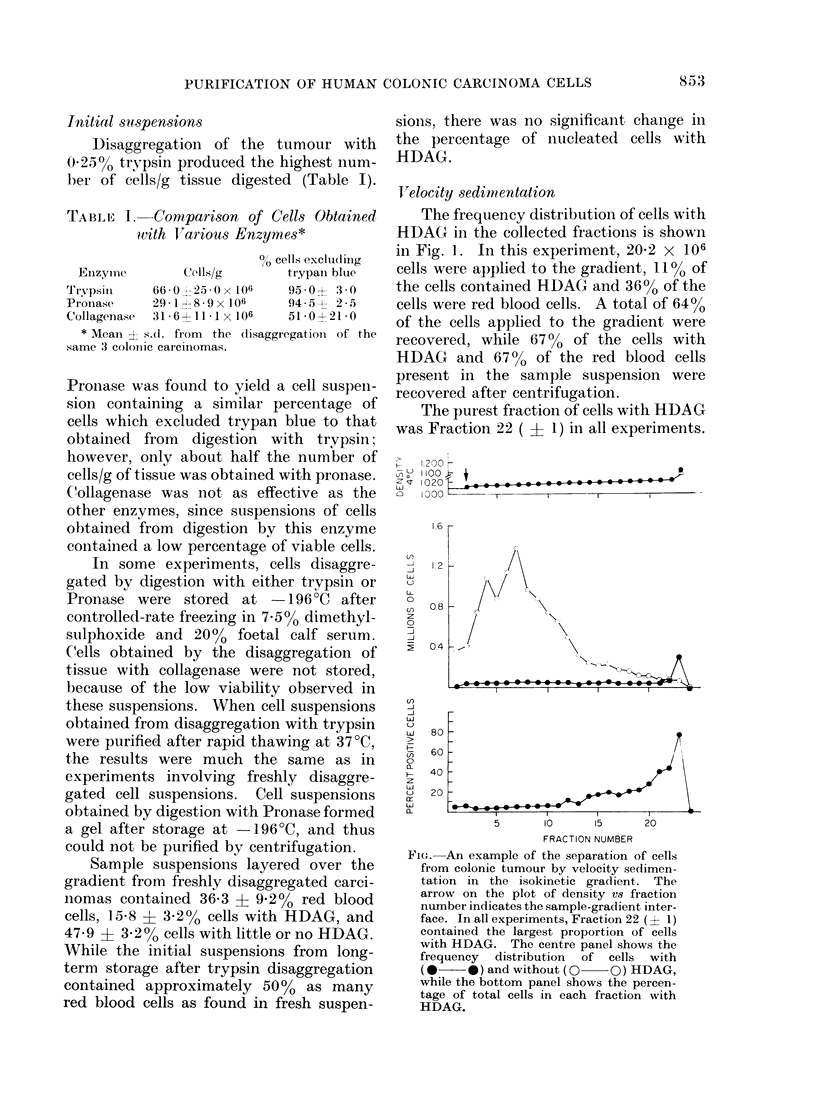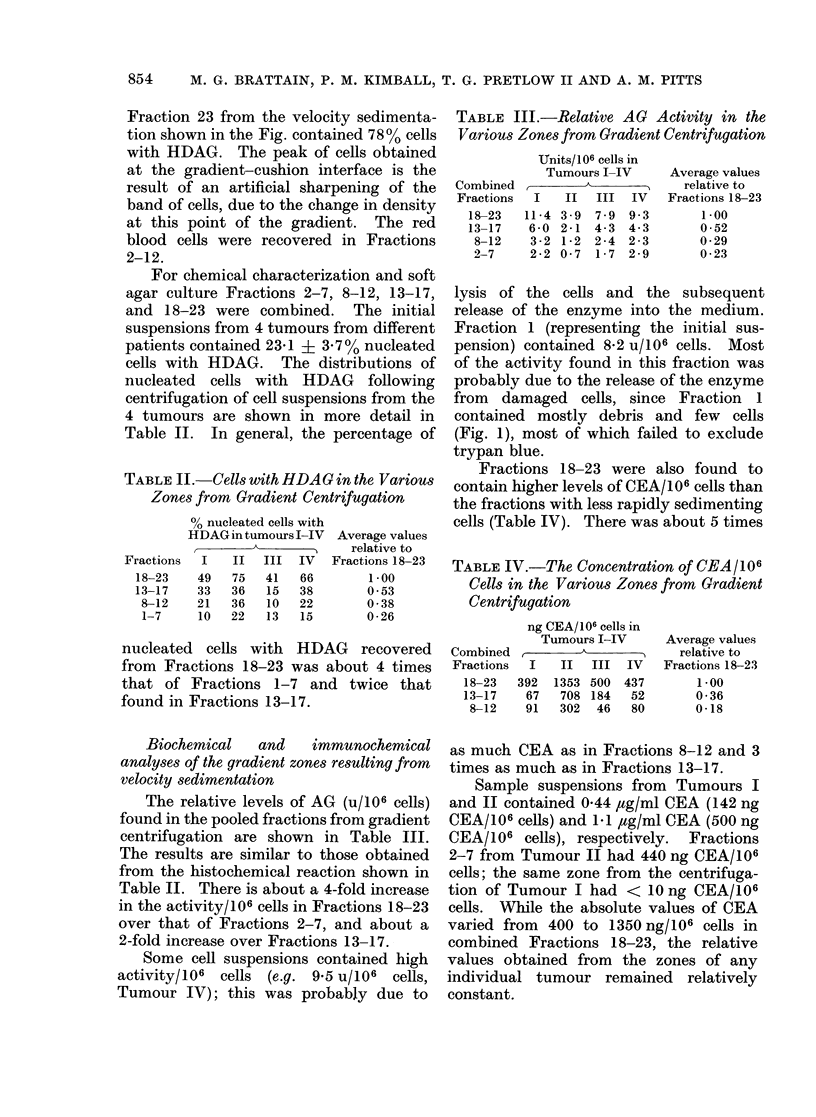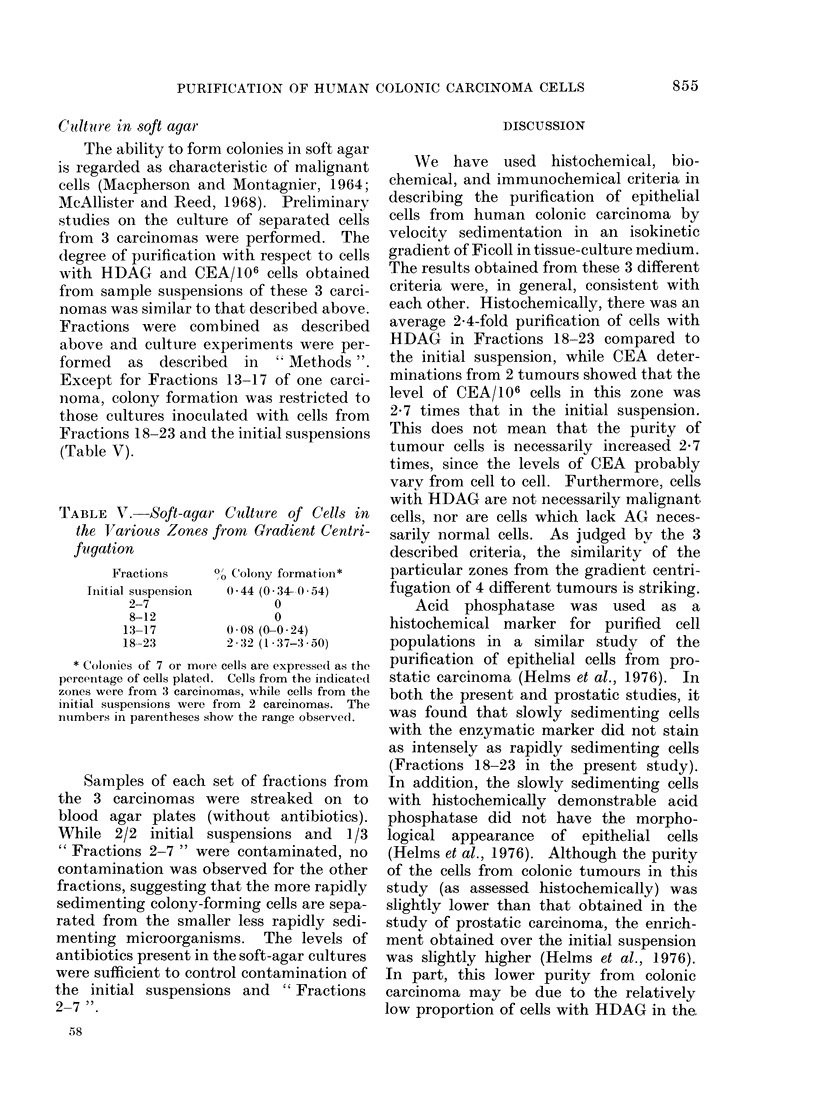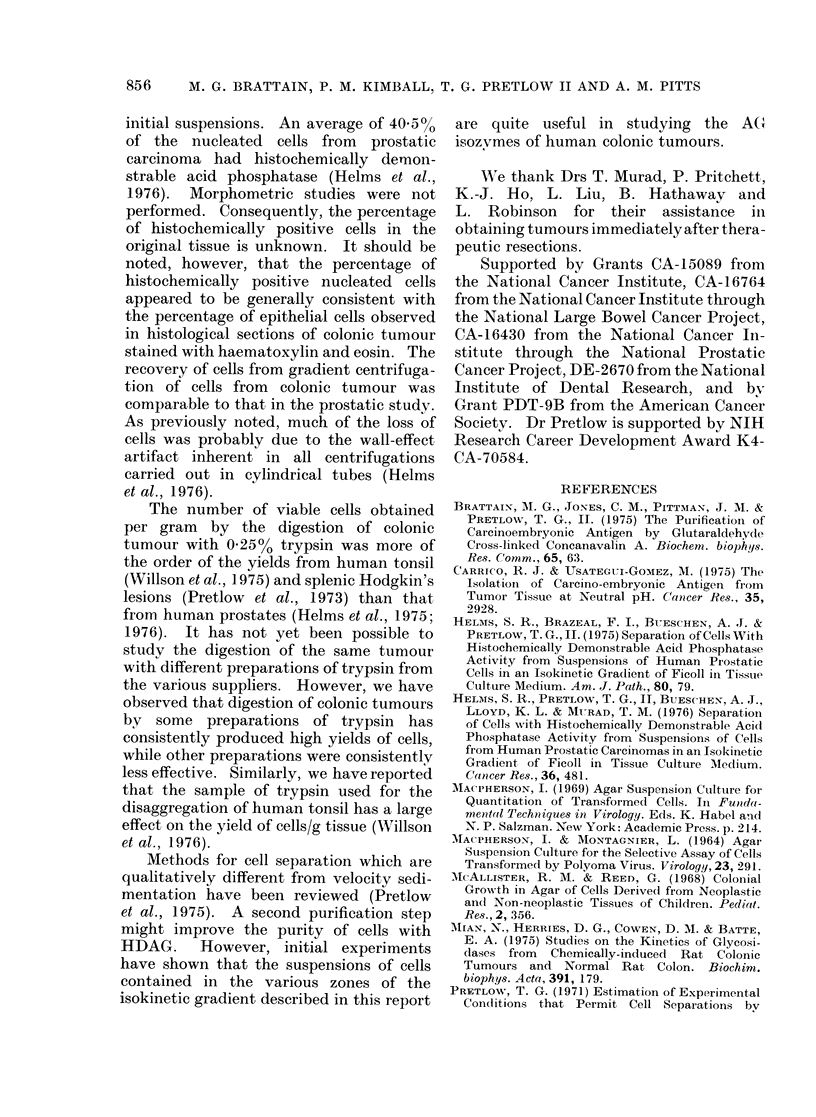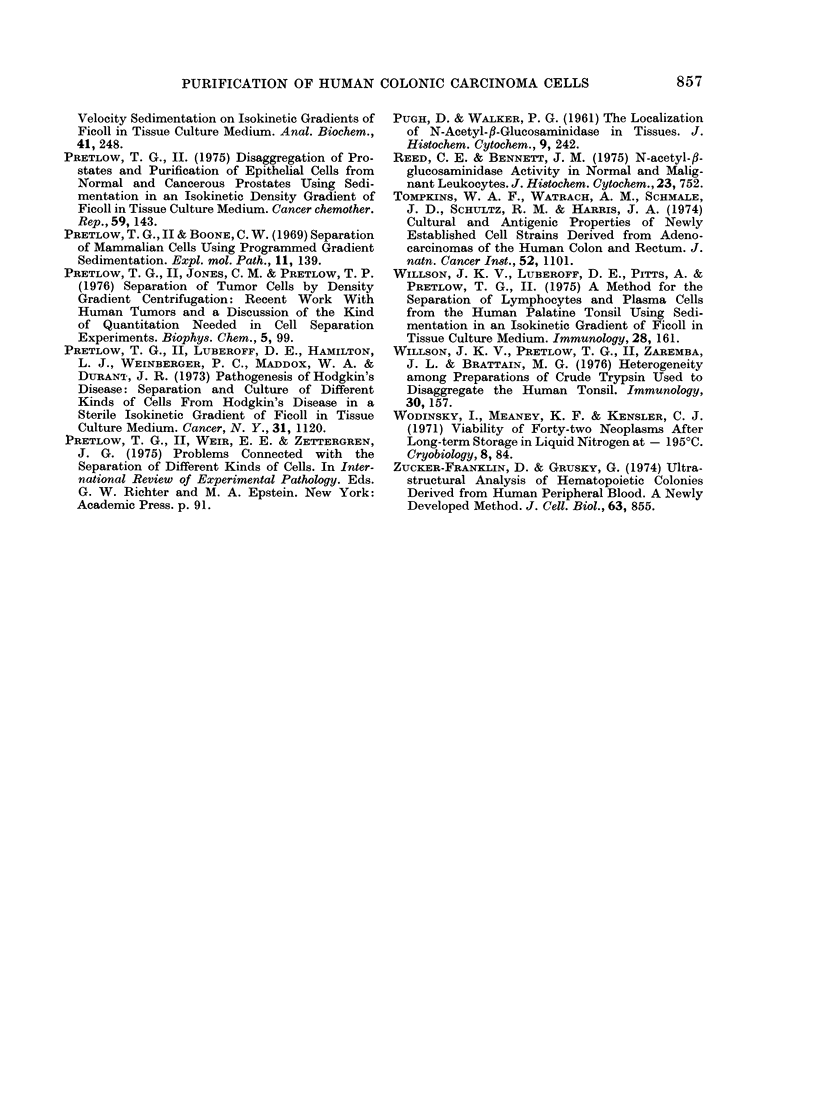# Partial purification of human colonic carcinoma cells by sedimentation.

**DOI:** 10.1038/bjc.1977.128

**Published:** 1977-06

**Authors:** M. G. Brattain, P. M. Kimball, T. G. Pretlow, A. M. Pitts

## Abstract

We have purified epithelial cells from human colonic tumours by velocity sedimentation in an isokinetic density gradient of Ficoll in tissue culture medium. In frozen sections of colonic carcinoma, histochemically demonstrable N-acetyl-beta-D-glucosaminidase (HDAG) was observed primarily in epithelial cells. We used this enzyme as a histochemical marker of epithelial cells. Initial suspensions of cells from colonic tumours suspended with 0-25% trypsin contained an average of 24% of the nucleated cells with HDAG. In the purest fraction obtained from gradient centrifugations, an average of 74% of the nucleated cells contained HDAG. After centrifugation, the quarter of the density gradient which contained the most rapidly sedimenting cells was purified 2-4-fold over that in the initial suspension. Cells in this zone of the gradient also gave rise to colonies in soft agar. Cells from initial suspension resulted in 15-25% as many colonies of 7 or more cells in cultures inoculated with the same number of nucleated cells. For the most part, cells obtained from the other zones of the gradient did not give rise to colonies in soft agar.


*Br. J. Cancer* (1977) **35**, 850.

### PARTIAL PURIFICATION OF HUMAN COLONIC CARCINOMA CELLS BY SEDIMENTATION

M. G. BRATTAIN, P. M. KIMBALL, T. G. PRETLOW II, AND A. M. PITTS

*From the Departments of Pathology and Biochemistry, University of Alabama Medical Centre, Birmingham, Alabama, U.S.A.* 35294

Received 7 October 1976 Accepted 14 February 1977

**Summary.—We have purified epithelial cells from human colonic tumours by velocity sedimentation in an isokinetic density gradient of Ficoll in tissue culture medium. In frozen sections of colonic carcinoma, histochemically demonstrable N-acetyl-β-D-glucosaminidase (HDAG) was observed primarily in epithelial cells. We used this enzyme as a histochemical marker of epithelial cells. Initial suspensions of cells from colonic tumours suspended with 0·25% trypsin contained an average of 24% of the nucleated cells with HDAG. In the purest fraction obtained from gradient centrifugations, an average of 74% of the nucleated cells contained HDAG. After centrifugation, the quarter of the density gradient which contained the most rapidly sedimenting cells was purified 2·4-fold over the initial sample with respect to nucleated cells with HDAG. The amount of carcinoembryonic antigen/$10^6$ cells in this zone of the gradient was increased 2·7-fold over that in the initial suspension. Cells in this zone of the gradient also gave rise to colonies in soft agar. Cells from initial suspensions resulted in 15–25% as many colonies of 7 or more cells in cultures inoculated with the same number of nucleated cells. For the most part, cells obtained from the other zones of the gradient did not give rise to colonies in soft agar.**

COMPARISONS of normal tissues and carcinomas are made difficult by the fact that the tumour cells in carcinomas and their epithelial counterparts in normal tissues often constitute a minority of the total population of cells (Helms *et al.*, 1975; Pretlow, 1975; Pretlow, Jones and Pretlow, 1976). If the cell types of interest are present in a low concentration, important biochemical differences among the various types of cells may be obscured. The comparison of malignant cells with other types of cells would be facilitated by the separation of these cells from the other kinds of cells with which they are associated in tissue (Pretlow *et al.*, 1976).

We have developed a method for the purification of epithelial cells from human colonic carcinomas, by centrifugation in a previously described isokinetic density gradient of Ficoll (polysucrose, average mol. wt. 400,000) in tissue culture medium (Pretlow, 1971). The histochemical demonstration of N-acetyl-β-D-glucosaminidase and culture in soft agar were used to identify purified cells.

### METHODS

*Demonstration of N-acetyl-β-D-glucosaminidase.*—N-acetyl-β-D-glucosaminidase (AG) was demonstrated histochemically by a slight modification of the diazo-coupling method described by Pugh and Walker (1961) using naphthol-AS-Bi-N-acetyl-β-D-glucosaminide (Calbiochem, La Jolla, Calif., U.S.A.) as substrate. Frozen sections of colonic tumour (10 μm) were incubated with the substrate at 37°C for 2–24 h. At this temperature, incubations of 4 h resulted in the greatest contrast between epithelial and non-epithelial components of the sections. Sections were counterstained with 1% fast

Address reprint requests to Dr Michael G. Brattain, Department of Pathology, University of Alabama Medical Center, Box 189, University Station, Birmingham, Alabama 35294.

green for 10 min. Histochemistry of cell suspensions was performed on cells prepared with the cytocentrifuge (Shandon Southern Instruments, Inc., Sewickley, Pa., U.S.A.); incubations were for 1–2 h at 37°C.

*Density gradient and centrifugation.*—A two-chambered gradient generator (Lido Glass, Stirling, N.J., U.S.A.) was utilized in the construction of density gradients of Ficoll (Pharmacia Fine Chemicals, Inc., Piscataway, N.J., U.S.A.) in tissue-culture medium, as described previously (Pretlow and Boone, 1969). Purification of colonic epithelial cells was obtained by velocity sedimentation in the 85-ml isokinetic gradient. The isokinetic gradient, which has been described in detail (Pretlow, 1971), was constructed in a 100-ml polycarbonate centrifuge tube (Tube No. 2806, International Equipment Co., Needham Heights, Mass., U.S.A.) and centrifuged in the MSE Mistral 6L centrifuge (Measuring and Scientific Equipment, Ltd, London, England). The gradient varies from 2·7% w/w Ficoll at the sample–gradient interface (13·7 cm from the centre of revolution) to 5·5% w/w Ficoll at the gradient–cushion interface (26·7 cm from the centre of revolution). The isokinetic gradient (in which cells sediment with constant velocity) is most useful for the separation of cells with different diameters (Pretlow, Weir and Zettergren, 1975). It was determined by previously described methods (Pretlow, 1971; Pretlow *et al.*, 1975) that cells with histochemically demonstrable N-acetyl-β-D-glucosaminidase (HDAG) and the highest concentration of carcinoembryonic antigen (CEA) per cell were best separated from other cells by centrifugation at 74 *g* (measured at the sample–gradient interface) for 10 min at 4°C. The centrifuge speed was monitored by an electronic stroboscope (General Radio Co., West Concord, Mass., U.S.A.).

*Cells for initial suspension.*—Colonic tumours were obtained by surgery at the University Hospital, Veterans Administration Hospital, and St Vincent's Hospital in Birmingham, Ala., U.S.A. Tissue was immediately placed in cold tissue-culture medium and subsequently minced in cold tissue-culture medium containing 10% foetal calf serum. After the tissue fragments were allowed to settle, the tissue-culture medium containing 10% foetal calf serum was decanted, and fresh medium was added and gently stirred with a magnetic stirrer for 10 min at room temperature. The tissue fragments were washed for two additional 10-min periods in this manner and then resuspended in tissue-culture medium containing 0·1% pronase (EM Laboratories, Inc., Elmsford, N.Y., U.S.A.), 0·25% trypsin (Microbiological Associates, Bethesda, Md., U.S.A.), or 0·05% collagenase (Sigma Chemical Co., St Louis Mo., U.S.A.). Enzymatic digestion of the tissue was performed in parallel with each of the three enzymes for 15 successive 20-min periods, to determine which enzyme would give the largest number of cells per gram of tissue; viability was determined by the exclusion of trypan blue. The cells from each of the 20-min periods of digestion were decanted from the tissue fragments, cooled in an ice bath for 5 min, and sedimented at 97 *g* for 7·5 min. The cells were resuspended in 5 volumes of medium containing 10% foetal calf serum and stored in an ice bath until the tissue was exhaustively digested. The first 3 digestions were discarded, as they contained many red blood cells and much debris. The remaining digestions were filtered through a single layer of Nitex (TETKO, Inc., Elmsford, N.Y., U.S.A.) having a pore diameter of 100 μm. Initial suspensions obtained in this manner contained $22{\cdot}4\text{–}33{\cdot}7 \times 10^6$ cells in the 7-ml volumes that were layered over the gradients.

Several experiments were performed with cells which had been stored at − 196°C over liquid $N_2$ for approximately one year after controlled-rate freezing at 1–2°C per min in 7·5% dimethylsulphoxide and 20% foetal calf serum in tissue-culture medium as described for cells from Hodgkin's disease (Pretlow *et al.*, 1973). Little if any change in viability has been observed for cells from tumours stored in this way (Pretlow *et al.*, 1973; Wodinsky, Meaney and Kensler, 1971). Cell suspensions were prepared for centrifugation by rapidly thawing the frozen cells at 37°C and diluting the suspension with an equal volume of tissue-culture medium. Sample suspensions prepared in this manner contained $12{\cdot}6\text{–}21{\cdot}7 \times 10^6$ cells in the 7-ml volumes layered over the gradients.

*Gradient fractions.*—The gradients were collected in 4-ml fractions (except for the first fraction representing the 7-ml initial volume) with a gradient-tapping cap (Halpro, Inc., Rockville, Md., U.S.A.) as described previously (Pretlow *et al.*, 1975). The refractive indices of the fractions were measured with an Abbe refractometer (Arthur H. Thomas Co., Philadelphia, Pa., U.S.A.). The cells in each fraction were counted in haemocytometer chambers. Slides for Wright's stain and N-acetyl-β-D-glucosaminidase were prepared with the cytocentrifuge. Differential cell counts were performed counting 500 cells from sample slides and 200 cells from each fraction.

*N-acetyl-β-D-glucosaminidase assay.*—The levels of N-acetyl-β-D-glucosaminidase (AG) in combined fractions from the gradients were assayed spectrophotometrically by measuring the release of p-nitrophenol from p-nitrophenyl-N-acetyl-β-D-glucosaminide (Sigma Chemical Co.). The combined fractions from 3 gradients (cells obtained from the same patient) were diluted to a refractive index of approximately 4·0 and recovered by centrifugation at 97 *g* for 7·5 min at 4°C. The cells were resuspended in 2–4 ml cold phosphate-buffered saline (0·01M, pH 7·2) and counted in haemocytometer chambers. Triton X-100 (final concentration 0·1%) was added to the cell suspension and the suspensions were sonicated by a Bronwill sonicator at 70% of the low energy setting for 1 min. Final cell concentrations ranged from $0{\cdot}1 \times 10^6$ cells/ml (Fractions 18–23) to $2{\cdot}4 \times 10^6$ cells/ml (Fractions 2–7). The level of enzymatic activity was found to be quite variable from patient to patient; consequently, the time of incubation and the amount of sample assayed were varied from 1 to 3 h and 0·1 to 0·3 ml of sample, respectively.

Assay mixtures contained 4 μmol of substrate and 50 μmol of citrate buffer in a final volume of 1·0 ml (pH 4·5). Controls were lacking either the enzyme sample or the substrate, and the values obtained from both controls were taken into account in the calculations of enzymatic activity. Reactions were terminated by the addition of 2 ml of 0·4 M glycine buffer (pH 10·4). The amount of p-nitrophenol released was calculated from a standard curve of commercial p-nitrophenol (Sigma Chemical Co.) determined spectrophotometrically at 410 nm. One unit of activity is defined as the release of 1 nmol of p-nitrophenol/min.

*Determination of CEA.*—Cell suspensions were prepared as described above for the spectrophotometric determination except that Triton X-100 was not added to the suspension prior to sonication. A recent study has shown sonication to be an effective means of extracting the CEA present in tissue homogenates (Carrico and Usategui-Gomez, 1975). CEA was determined by the commercially available radioimmunoassay (Roche Diagnostics, Nutley, N.J., U.S.A.) as described previously (Brattain *et al.*, 1975).

*Culture in soft agar.*—Carcinomas from 3 additional patients were disaggregated and stored at −196°C after controlled-rate freezing as described above, except that sterile procedure was observed after obtaining the specimen. Cells from these stored samples were purified as described above in a sterile isokinetic density gradient and sterile technique was followed for the collection of gradient fractions. Cells (50,000, nucleated) were cultured on plastic Petri dishes in a final volume of 1 ml of 0·27% soft agar containing 4·3 μg/ml gentamycin, 90 μg/ml streptomycin and 90 u/ml penicillin, on a 1-ml layer of 0·5% soft agar containing the same levels of antibiotics (Macpherson, 1969; Tompkins *et al.*, 1974). The colonies were counted microscopically after embedding the entire contents of the Petri dish in Epon as described by Zucker-Franklin and Grusky (1974). Preliminary studies showed that colonies were maximally developed after 3–4 weeks. Colonies were counted after one month of culture, and a colony was defined as containing 7 or more cells. Controls for each set of fractions and the sample suspensions were embedded and counted at the time of plating.

### RESULTS

### *N-acetyl-β-D-glucosaminidase activity in frozen sections of colonic tumour*

It was found that the epithelial regions in frozen sections of colonic tumour showed stronger staining for AG than the stroma. Raised AG activity has also been observed by histochemical means in circulating monocytes from patients with malignant solid tumours (Reed and Bennett, 1975). The staining could be eliminated by adding 100 mM N-acetyl-glucosamine to the substrate solution after pre-incubation of the sections for 30 min at 37°C in 100 mM N-acetyl-glucosamine. Pre-incubation in buffer alone did not alter the staining of frozen sections. N-acetyl-glucosamine is a competitive inhibitor of the enzyme (Mian *et al.*, 1975).

*Initial suspensions*

Disaggregation of the tumour with 0·25% trypsin produced the highest number of cells/g tissue digested (Table I).

TABLE I.—*Comparison of Cells Obtained with Various Enzymes**

| Enzyme | Cells/g | % cells excluding trypan blue |
|---|---|---|
| Trypsin | $66·0 \pm 25·0 \times 10^6$ | $95·0 \pm 3·0$ |
| Pronase | $29·1 \pm 8·9 \times 10^6$ | $94·5 \pm 2·5$ |
| Collagenase | $31·6 \pm 11·1 \times 10^6$ | $51·0 \pm 21·0$ |

* Mean ± s.d. from the disaggregation of the same 3 colonic carcinomas.

Pronase was found to yield a cell suspension containing a similar percentage of cells which excluded trypan blue to that obtained from digestion with trypsin; however, only about half the number of cells/g of tissue was obtained with pronase. Collagenase was not as effective as the other enzymes, since suspensions of cells obtained from digestion by this enzyme contained a low percentage of viable cells.

In some experiments, cells disaggregated by digestion with either trypsin or Pronase were stored at −196°C after controlled-rate freezing in 7·5% dimethylsulphoxide and 20% foetal calf serum. Cells obtained by the disaggregation of tissue with collagenase were not stored, because of the low viability observed in these suspensions. When cell suspensions obtained from disaggregation with trypsin were purified after rapid thawing at 37°C, the results were much the same as in experiments involving freshly disaggregated cell suspensions. Cell suspensions obtained by digestion with Pronase formed a gel after storage at −196°C, and thus could not be purified by centrifugation.

Sample suspensions layered over the gradient from freshly disaggregated carcinomas contained 36·3 ± 9·2% red blood cells, 15·8 ± 3·2% cells with HDAG, and 47·9 ± 3·2% cells with little or no HDAG. While the initial suspensions from long-term storage after trypsin disaggregation contained approximately 50% as many red blood cells as found in fresh suspensions, there was no significant change in the percentage of nucleated cells with HDAG.

*Velocity sedimentation*

The frequency distribution of cells with HDAG in the collected fractions is shown in Fig. 1. In this experiment, $20·2 \times 10^6$ cells were applied to the gradient, 11% of the cells contained HDAG and 36% of the cells were red blood cells. A total of 64% of the cells applied to the gradient were recovered, while 67% of the cells with HDAG and 67% of the red blood cells present in the sample suspension were recovered after centrifugation.

The purest fraction of cells with HDAG was Fraction 22 ( ± 1) in all experiments.

FIG.—An example of the separation of cells from colonic tumour by velocity sedimentation in the isokinetic gradient. The arrow on the plot of density *vs* fraction number indicates the sample-gradient interface. In all experiments, Fraction 22 (± 1) contained the largest proportion of cells with HDAG. The centre panel shows the frequency distribution of cells with (●——●) and without (○——○) HDAG, while the bottom panel shows the percentage of total cells in each fraction with HDAG.

Fraction 23 from the velocity sedimentation shown in the Fig. contained 78% cells with HDAG. The peak of cells obtained at the gradient–cushion interface is the result of an artificial sharpening of the band of cells, due to the change in density at this point of the gradient. The red blood cells were recovered in Fractions 2–12.

For chemical characterization and soft agar culture Fractions 2–7, 8–12, 13–17, and 18–23 were combined. The initial suspensions from 4 tumours from different patients contained 23·1 ± 3·7% nucleated cells with HDAG. The distributions of nucleated cells with HDAG following centrifugation of cell suspensions from the 4 tumours are shown in more detail in Table II. In general, the percentage of

TABLE II.—*Cells with HDAG in the Various Zones from Gradient Centrifugation*

| Fractions | % nucleated cells with HDAG in tumours I–IV: I | II | III | IV | Average values relative to Fractions 18–23 |
|---|---|---|---|---|---|
| 18–23 | 49 | 75 | 41 | 66 | 1·00 |
| 13–17 | 33 | 36 | 15 | 38 | 0·53 |
| 8–12 | 21 | 36 | 10 | 22 | 0·38 |
| 1–7 | 10 | 22 | 13 | 15 | 0·26 |

nucleated cells with HDAG recovered from Fractions 18–23 was about 4 times that of Fractions 1–7 and twice that found in Fractions 13–17.

*Biochemical and immunochemical analyses of the gradient zones resulting from velocity sedimentation*

The relative levels of AG (u/10⁶ cells) found in the pooled fractions from gradient centrifugation are shown in Table III. The results are similar to those obtained from the histochemical reaction shown in Table II. There is about a 4-fold increase in the activity/$10^6$ cells in Fractions 18–23 over that of Fractions 2–7, and about a 2-fold increase over Fractions 13–17.

Some cell suspensions contained high activity/$10^6$ cells (*e.g.* 9·5 u/$10^6$ cells, Tumour IV); this was probably due to

TABLE III.—*Relative AG Activity in the Various Zones from Gradient Centrifugation*

| Combined Fractions | Units/$10^6$ cells in Tumours I–IV: I | II | III | IV | Average values relative to Fractions 18–23 |
|---|---|---|---|---|---|
| 18–23 | 11·4 | 3·9 | 7·9 | 9·3 | 1·00 |
| 13–17 | 6·0 | 2·1 | 4·3 | 4·3 | 0·52 |
| 8–12 | 3·2 | 1·2 | 2·4 | 2·3 | 0·29 |
| 2–7 | 2·2 | 0·7 | 1·7 | 2·9 | 0·23 |

lysis of the cells and the subsequent release of the enzyme into the medium. Fraction 1 (representing the initial suspension) contained 8·2 u/$10^6$ cells. Most of the activity found in this fraction was probably due to the release of the enzyme from damaged cells, since Fraction 1 contained mostly debris and few cells (Fig. 1), most of which failed to exclude trypan blue.

Fractions 18–23 were also found to contain higher levels of CEA/$10^6$ cells than the fractions with less rapidly sedimenting cells (Table IV). There was about 5 times

TABLE IV.—*The Concentration of CEA/$10^6$ Cells in the Various Zones from Gradient Centrifugation*

| Combined Fractions | ng CEA/$10^6$ cells in Tumours I–IV: I | II | III | IV | Average values relative to Fractions 18–23 |
|---|---|---|---|---|---|
| 18–23 | 392 | 1353 | 500 | 437 | 1·00 |
| 13–17 | 67 | 708 | 184 | 52 | 0·36 |
| 8–12 | 91 | 302 | 46 | 80 | 0·18 |

as much CEA as in Fractions 8–12 and 3 times as much as in Fractions 13–17.

Sample suspensions from Tumours I and II contained 0·44 μg/ml CEA (142 ng CEA/$10^6$ cells) and 1·1 μg/ml CEA (500 ng CEA/$10^6$ cells), respectively. Fractions 2–7 from Tumour II had 440 ng CEA/$10^6$ cells; the same zone from the centrifugation of Tumour I had < 10 ng CEA/$10^6$ cells. While the absolute values of CEA varied from 400 to 1350 ng/$10^6$ cells in combined Fractions 18–23, the relative values obtained from the zones of any individual tumour remained relatively constant.

*Culture in soft agar*

The ability to form colonies in soft agar is regarded as characteristic of malignant cells (Macpherson and Montagnier, 1964; McAllister and Reed, 1968). Preliminary studies on the culture of separated cells from 3 carcinomas were performed. The degree of purification with respect to cells with HDAG and CEA/$10^6$ cells obtained from sample suspensions of these 3 carcinomas was similar to that described above. Fractions were combined as described above and culture experiments were performed as described in " Methods ". Except for Fractions 13–17 of one carcinoma, colony formation was restricted to those cultures inoculated with cells from Fractions 18–23 and the initial suspensions (Table V).

TABLE V.—*Soft-agar Culture of Cells in the Various Zones from Gradient Centrifugation*

| Fractions | % Colony formation* |
|---|---|
| Initial suspension | 0·44 (0·34–0·54) |
| 2–7 | 0 |
| 8–12 | 0 |
| 13–17 | 0·08 (0–0·24) |
| 18–23 | 2·32 (1·37–3·50) |

* Colonies of 7 or more cells are expressed as the percentage of cells plated. Cells from the indicated zones were from 3 carcinomas, while cells from the initial suspensions were from 2 carcinomas. The numbers in parentheses show the range observed.

Samples of each set of fractions from the 3 carcinomas were streaked on to blood agar plates (without antibiotics). While 2/2 initial suspensions and 1/3 " Fractions 2–7 " were contaminated, no contamination was observed for the other fractions, suggesting that the more rapidly sedimenting colony-forming cells are separated from the smaller less rapidly sedimenting microorganisms. The levels of antibiotics present in the soft-agar cultures were sufficient to control contamination of the initial suspensions and " Fractions 2–7 ".

### DISCUSSION

We have used histochemical, biochemical, and immunochemical criteria in describing the purification of epithelial cells from human colonic carcinoma by velocity sedimentation in an isokinetic gradient of Ficoll in tissue-culture medium. The results obtained from these 3 different criteria were, in general, consistent with each other. Histochemically, there was an average 2·4-fold purification of cells with HDAG in Fractions 18–23 compared to the initial suspension, while CEA determinations from 2 tumours showed that the level of CEA/$10^6$ cells in this zone was 2·7 times that in the initial suspension. This does not mean that the purity of tumour cells is necessarily increased 2·7 times, since the levels of CEA probably vary from cell to cell. Furthermore, cells with HDAG are not necessarily malignant cells, nor are cells which lack AG necessarily normal cells. As judged by the 3 described criteria, the similarity of the particular zones from the gradient centrifugation of 4 different tumours is striking.

Acid phosphatase was used as a histochemical marker for purified cell populations in a similar study of the purification of epithelial cells from prostatic carcinoma (Helms *et al.*, 1976). In both the present and prostatic studies, it was found that slowly sedimenting cells with the enzymatic marker did not stain as intensely as rapidly sedimenting cells (Fractions 18–23 in the present study). In addition, the slowly sedimenting cells with histochemically demonstrable acid phosphatase did not have the morphological appearance of epithelial cells (Helms *et al.*, 1976). Although the purity of the cells from colonic tumours in this study (as assessed histochemically) was slightly lower than that obtained in the study of prostatic carcinoma, the enrichment obtained over the initial suspension was slightly higher (Helms *et al.*, 1976). In part, this lower purity from colonic carcinoma may be due to the relatively low proportion of cells with HDAG in the initial suspensions. An average of 40·5% of the nucleated cells from prostatic carcinoma had histochemically demonstrable acid phosphatase (Helms *et al.*, 1976). Morphometric studies were not performed. Consequently, the percentage of histochemically positive cells in the original tissue is unknown. It should be noted, however, that the percentage of histochemically positive nucleated cells appeared to be generally consistent with the percentage of epithelial cells observed in histological sections of colonic tumour stained with haematoxylin and eosin. The recovery of cells from gradient centrifugation of cells from colonic tumour was comparable to that in the prostatic study. As previously noted, much of the loss of cells was probably due to the wall-effect artifact inherent in all centrifugations carried out in cylindrical tubes (Helms *et al.*, 1976).

The number of viable cells obtained per gram by the digestion of colonic tumour with 0·25% trypsin was more of the order of the yields from human tonsil (Willson *et al.*, 1975) and splenic Hodgkin's lesions (Pretlow *et al.*, 1973) than that from human prostates (Helms *et al.*, 1975; 1976). It has not yet been possible to study the digestion of the same tumour with different preparations of trypsin from the various suppliers. However, we have observed that digestion of colonic tumours by some preparations of trypsin has consistently produced high yields of cells, while other preparations were consistently less effective. Similarly, we have reported that the sample of trypsin used for the disaggregation of human tonsil has a large effect on the yield of cells/g tissue (Willson *et al.*, 1976).

Methods for cell separation which are qualitatively different from velocity sedimentation have been reviewed (Pretlow *et al.*, 1975). A second purification step might improve the purity of cells with HDAG. However, initial experiments have shown that the suspensions of cells contained in the various zones of the isokinetic gradient described in this report are quite useful in studying the AG isozymes of human colonic tumours.

We thank Drs T. Murad, P. Pritchett, K.-J. Ho, L. Liu, B. Hathaway and L. Robinson for their assistance in obtaining tumours immediately after therapeutic resections.

Supported by Grants CA-15089 from the National Cancer Institute, CA-16764 from the National Cancer Institute through the National Large Bowel Cancer Project, CA-16430 from the National Cancer Institute through the National Prostatic Cancer Project, DE-2670 from the National Institute of Dental Research, and by Grant PDT-9B from the American Cancer Society. Dr Pretlow is supported by NIH Research Career Development Award K4-CA-70584.

### REFERENCES

Brattain, M. G., Jones, C. M., Pittman, J. M. & Pretlow, T. G., II. (1975) The Purification of Carcinoembryonic Antigen by Glutaraldehyde Cross-linked Concanavalin A. *Biochem. biophys. Res. Comm.*, **65**, 63.

Carrico, R. J. & Usategui-Gomez, M. (1975) The Isolation of Carcino-embryonic Antigen from Tumor Tissue at Neutral pH. *Cancer Res.*, **35**, 2928.

Helms, S. R., Brazeal, F. I., Bueschen, A. J. & Pretlow, T. G., II. (1975) Separation of Cells With Histochemically Demonstrable Acid Phosphatase Activity from Suspensions of Human Prostatic Cells in an Isokinetic Gradient of Ficoll in Tissue Culture Medium. *Am. J. Path.*, **80**, 79.

Helms, S. R., Pretlow, T. G., II, Bueschen, A. J., Lloyd, K. L. & Murad, T. M. (1976) Separation of Cells with Histochemically Demonstrable Acid Phosphatase Activity from Suspensions of Cells from Human Prostatic Carcinomas in an Isokinetic Gradient of Ficoll in Tissue Culture Medium. *Cancer Res.*, **36**, 481.

Macpherson, I. (1969) Agar Suspension Culture for Quantitation of Transformed Cells. In *Fundamental Techniques in Virology*. Eds. K. Habel and N. P. Salzman. New York: Academic Press. p. 214.

Macpherson, I. & Montagnier, L. (1964) Agar Suspension Culture for the Selective Assay of Cells Transformed by Polyoma Virus. *Virology*, **23**, 291.

McAllister, R. M. & Reed, G. (1968) Colonial Growth in Agar of Cells Derived from Neoplastic and Non-neoplastic Tissues of Children. *Pediat. Res.*, **2**, 356.

Mian, N., Herries, D. G., Cowen, D. M. & Batte, E. A. (1975) Studies on the Kinetics of Glycosidases from Chemically-induced Rat Colonic Tumours and Normal Rat Colon. *Biochim. biophys. Acta*, **391**, 179.

Pretlow, T. G. (1971) Estimation of Experimental Conditions that Permit Cell Separations by

Velocity Sedimentation on Isokinetic Gradients of Ficoll in Tissue Culture Medium. *Anal. Biochem.*, **41**, 248.

PRETLOW, T. G., II. (1975) Disaggregation of Prostates and Purification of Epithelial Cells from Normal and Cancerous Prostates Using Sedimentation in an Isokinetic Density Gradient of Ficoll in Tissue Culture Medium. *Cancer chemother. Rep.*, **59**, 143.

PRETLOW, T. G., II & BOONE, C. W. (1969) Separation of Mammalian Cells Using Programmed Gradient Sedimentation. *Expl. mol. Path.*, **11**, 139.

PRETLOW, T. G., II, JONES, C. M. & PRETLOW, T. P. (1976) Separation of Tumor Cells by Density Gradient Centrifugation: Recent Work With Human Tumors and a Discussion of the Kind of Quantitation Needed in Cell Separation Experiments. *Biophys. Chem.*, **5**, 99.

PRETLOW, T. G., II, LUBEROFF, D. E., HAMILTON, L. J., WEINBERGER, P. C., MADDOX, W. A. & DURANT, J. R. (1973) Pathogenesis of Hodgkin's Disease: Separation and Culture of Different Kinds of Cells From Hodgkin's Disease in a Sterile Isokinetic Gradient of Ficoll in Tissue Culture Medium. *Cancer, N. Y.*, **31**, 1120.

PRETLOW, T. G., II, WEIR, E. E. & ZETTERGREN, J. G. (1975) Problems Connected with the Separation of Different Kinds of Cells. In *International Review of Experimental Pathology*. Eds. G. W. Richter and M. A. Epstein. New York: Academic Press. p. 91.

PUGH, D. & WALKER, P. G. (1961) The Localization of N-Acetyl-β-Glucosaminidase in Tissues. *J. Histochem. Cytochem.*, **9**, 242.

REED, C. E. & BENNETT, J. M. (1975) N-acetyl-β-glucosaminidase Activity in Normal and Malignant Leukocytes. *J. Histochem. Cytochem.*, **23**, 752.

TOMPKINS, W. A. F., WATRACH, A. M., SCHMALE, J. D., SCHULTZ, R. M. & HARRIS, J. A. (1974) Cultural and Antigenic Properties of Newly Established Cell Strains Derived from Adenocarcinomas of the Human Colon and Rectum. *J. natn. Cancer Inst.*, **52**, 1101.

WILLSON, J. K. V., LUBEROFF, D. E., PITTS, A. & PRETLOW, T. G., II. (1975) A Method for the Separation of Lymphocytes and Plasma Cells from the Human Palatine Tonsil Using Sedimentation in an Isokinetic Gradient of Ficoll in Tissue Culture Medium. *Immunology*, **28**, 161.

WILLSON, J. K. V., PRETLOW, T. G., II, ZAREMBA, J. L. & BRATTAIN, M. G. (1976) Heterogeneity among Preparations of Crude Trypsin Used to Disaggregate the Human Tonsil. *Immunology*, **30**, 157.

WODINSKY, I., MEANEY, K. F. & KENSLER, C. J. (1971) Viability of Forty-two Neoplasms After Long-term Storage in Liquid Nitrogen at − 195°C. *Cryobiology*, **8**, 84.

ZUCKER-FRANKLIN, D. & GRUSKY, G. (1974) Ultrastructural Analysis of Hematopoietic Colonies Derived from Human Peripheral Blood. A Newly Developed Method. *J. Cell. Biol.*, **63**, 855.